# 2736. Real-world Effectiveness of Posaconazole Prophylaxis in Preventing Invasive Fungal Infections in Recipients of Allogeneic Stem Cell Transplantation

**DOI:** 10.1093/ofid/ofad500.2347

**Published:** 2023-11-27

**Authors:** Carolina Álvarez-Ortega, Jenny Patricia Muñoz-Lombo, David E Rebellon-Sanchez, Julio Llanos-Torres, Fernando Rosso, Juan Diego Vélez, Pablo A Moncada

**Affiliations:** Fundacion Valle del Lili, Cali, Valle del Cauca, Colombia; Fundacion Valle del Lili, Cali, Valle del Cauca, Colombia; Fundacion Valle del Lili, Cali, Valle del Cauca, Colombia; Fundacion Valle del Lili, Cali, Valle del Cauca, Colombia; Fundacion Valle del Lili, Cali, Valle del Cauca, Colombia; Fundación Valle del Lili, cali, Valle del Cauca, Colombia; Fundación Valle del Lili, cali, Valle del Cauca, Colombia

## Abstract

**Background:**

Invasive fungal infections (IFIs) can cause high morbidity and mortality in immunocompromised patients. Posaconazole is a commonly used triazole antifungal agent for prophylaxis against IFIs in high-risk populations. However, the effectiveness of Posaconazole as antifungal prophylaxis in Allogeneic Stem Cell Transplantation (allo-SCT) in Latin-America has not been well described. The aim of this study is to describe the clinical experience with the use of Posaconazole as antifungal prophylaxis in allo-SCT in a high complexity medical center in Latin-America.

**Methods:**

A retrospective descriptive study was conducted to evaluate the experience of using Fluconazole or Posaconazole as antifungal prophylaxis in patients undergoing Allogeneic Stem Cell Transplant at the Fundación Valle de Lili in Cali, Colombia between 2012 and 2020. (Figure 1). The primary outcome variable was the incidence of IFI in the first 100 days of prophylaxis.

Allogenic Stem Cell Transplant: Methods selection

**Figure:**
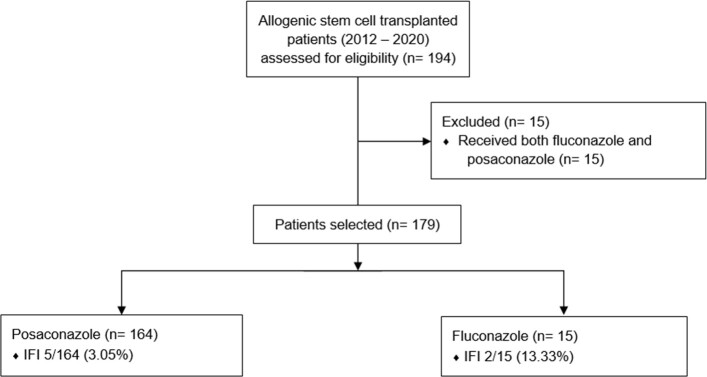

A Retrospective descriptive study involving the selection of 194 patients who underwent Allogeneic Stem Cell Transplantation, of which 179 patients met the selection criteria for the study.

**Results:**

This study analyzed 179 individuals with hematologic malignancies who received prophylaxis with either Posaconazole (n=164) or Fluconazole (n=15). The occurrence of IFI was lower in the Posaconazole group compared to the fluconazole group during the initial 100 days of prophylaxis (1.22% vs. 13.33%, p=0.03) (Figure 2). Aspergillus spp were the most frequent microorganisms observed in 3 cases (1.67%). The Number Needed to Treat (NNT) was 8.25, indicating that 8 patients treated with Posaconazole can prevent one IFI that would have occurred with Fluconazole treatment.

Comparative analysis of Posaconazole and Fluconazole as antifungal prophylaxis in Allogeneic Stem Cell Transplantation

**Figure:**
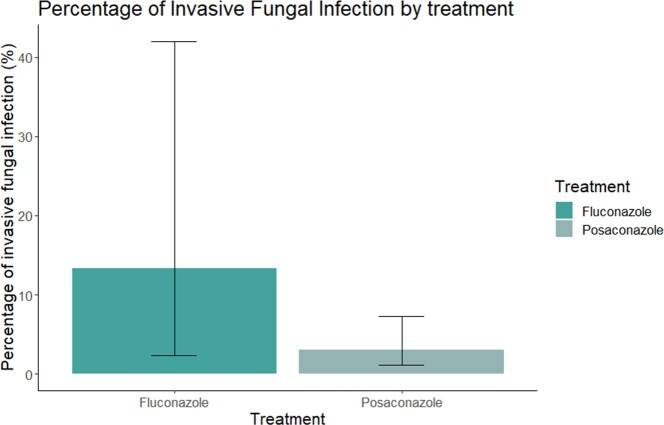

Percentage of Invasive Fungal Infections found in Allogeneic Stem Cell Transplantation during the first 100 days post-transplantation in patients who received prophylaxis with Posaconazole or Fluconazole.

**Conclusion:**

This study suggests that using Posaconazole as antifungal prophylaxis reduces the incidence of invasive fungal infections (IFI) in patients undergoing Allogenic Stem Cell Transplantation under real-world conditions and is a safe alternative to be used in low-income settings. Further clinical studies with larger sample sizes are required to confirm these findings.

**Disclosures:**

**All Authors**: No reported disclosures

